# Television Is Still “Easy” and Print Is Still “Tough”? More Than 30 Years of Research on the Amount of Invested Mental Effort

**DOI:** 10.3389/fpsyg.2018.01098

**Published:** 2018-07-03

**Authors:** Frank Schwab, Christine Hennighausen, Dorothea C. Adler, Astrid Carolus

**Affiliations:** Department of Media Psychology, University of Würzburg, Würzburg, Germany

**Keywords:** AIME, amount of invested mental effort, literature review, content-analysis, content cluster

## Abstract

We provide a literature overview of 30 years of research on the amount of invested mental effort (AIME, Salomon, 1984), illuminating relevant literature in this field. Since the introduction of AIME, this concept appears to have vanished. To obtain a clearer picture of where the theory of AIME has diffused, we conducted a literature search focusing on the period 1985–2015. We examined scientific articles (*N* = 244) that cite Salomon (1984) and content-analyzed their keywords. Based on these keywords, we identified seven content clusters: *affect and motivation*, *application fields*, *cognition and learning*, *education and teaching*, *media technology, learning with media technology*, and *methods*. We present selected works of each content cluster and describe in which research field the articles had been published. Results indicate that AIME was most commonly (but not exclusively) referred to in the area of educational psychology indicating its importance regarding learning and education, thereby investigating print and TV, as well as new media. From a methodological perspective, research applied various research methods (e.g., longitudinal studies, experimental designs, theoretical analysis) and samples (e.g., children, college students, low income families). From these findings, the importance of AIME for further research is discussed.

## Introduction

*Television is* “*easy*” *and print is* “*tough*” – this is the title of Salomon’s (1984) presumably most influential article on the AIME. In this research, Salomon investigated how much mental effort school children invested in learning from television (TV) as opposed to learning from print (i.e., a book). The results showed that children perceived TV as a rather easy medium, while they evaluated print as a rather difficult medium. This perception directly affected school children’s AIME, namely when they perceived a medium as easy they spent less effort on learning contents from it and scored lower on a subsequent achievement test. Strikingly, the children reported a higher perceived SE. In contrast, when children perceived a medium as tough they reported lower SE, were thus willing to invest higher mental effort in learning and achieved higher performance in a subsequent knowledge test. Salomon’s findings, however, could not be fully replicated. A Dutch study by Beentjes (1989) suggested that children’s AIME was rather content-depended so that it could not be generalized to a specific medium. The publications of these partially controversial findings are more than 30 years ago. Today, the concept of AIME as proposed by Salomon (1981, 1983a,b, 1984), Salomon and Leigh (1984) is rarely found in media and educational psychology research anymore. However, technology as well as media and media usage have changed dramatically since then. While research on AIME shed light on learning with media from the 1970s, the question arises to which research areas and theoretical concepts AIME has diffused in the last 30 years since the publication of Salomon’s (1984) article.

In the present article, we aim to approach this research question by providing an overview of past and current research that is related to AIME. To this end, we conducted a literature search in October 2015 using the database Web of Science^®^ (WoS) and selected journal articles from the years 1985 to 2015 that cite Salomon (1984). By content-analyzing the keywords of these articles, we identified seven content clusters. We describe each of them and hence show the reader where AIME has diffused. Finally, we discuss our findings and point at potential research fields that might benefit from AIME in the future.

### The Theory of AIME

Amount of invested mental effort is defined as the “number of non-automatic elaborations applied to material measured by learners’ self-reports” (Salomon, 1984, p. 647). According to Salomon (1984), when a specific instruction is absent, an individual’s AIME is predominantly influenced by two factors. The first factor concerns *the* PDC of the stimulus, the task, or the context. In line with Salomon (1984), more AIME will be invested when the PDC are perceived as demanding. For example, children learn more from TV when they are instructed to do so (i.e., task; Kwaitek and Watkins, 1981) and when they watch it together with their parents (i.e., context; Salomon, 1977). Moreover, when children perceive the medium as low demanding (i.e., stimulus, “image”), they invest less mental effort and learn less (Salomon, 1984). In particular, TV appears to be regarded as a medium that requires less mental effort relative to print due to its more lifelike and pictorial character (Salomon, 1981).

The second factor refers to PSE (Bandura, 1982) which describes the subjective assumption of how effectively one feels to be able to cognitively deal with the medium and learn something from it. Thereby, PSE and AIME are positively related when a task is perceived as difficult. However, when a task is perceived as easy, individuals may also expend less AIME because they assume that they can easily solve the task (Salomon, 1984).

Perceived demand characteristics (PDC; of the task, stimulus, or context) and PSE are related to each other and both affect AIME; AIME, in turn, influences learning.

Salomon (1984) suggested that PDC and PSE influence AIME when specific, explicit, or unambiguous task instructions are missing. Specifically, he argued that PDC and PSE would interact with each other such that AIME is high either when both PDC and PSE are low, or when they are both high. In contrast, when one of the factors is high while the other is low, low AIME will be invested.

Cennamo (1993) reviewed the literature on AIME and learning from video and focused on the factors determining the learner’s preconception. She concluded that three types of characteristics influence preconceptions toward a specific medium, which, in turn affect AIME and eventually learning: (a) *perceived characteristics of the stimulus* (e.g., differences in the symbol systems leading to the perception that TV is easier than print), (b) the *task* (e.g., educational vs. entertainment materials, specific contents), and (c) *learner characteristics* (e.g., age, experience with medium, media preferences).

### The Beginning: AIME Research in the 1970s, 1980s, and 1990s

Salomon started investigating AIME in the 1970s. In one of his first studies, Cohen and Salomon (1979) investigated the effect of television watching on the development of specific mental skills. The authors conducted a cross-cultural study involving American middle-class children (heavy TV viewers) and Israeli middle-class children (lighter TV viewers). They measured the children’s TV consumption and the amount of literate viewing, meaning concentrative and comprehensive viewing. Results showed that Israeli children scored higher on the literate viewing measure while consuming less TV than the American children. The authors explained this unexpected finding by the specific characteristics of TV: its symbol system and fast-paced presentation of information leaves little time for mental elaboration only (Singer, 1980). Cohen and Salomon further suggested that demand characteristics of the task (i.e., an instruction to actively remember information presented in TV), motivation, characteristics of the situation, and prevailing social norms relating to the televiewing situation would influence the depth of information processing from TV.

In his central study, Salomon (1984) examined the relationships between learning, AIME, and type of medium. Salomon assessed sixth graders’ perceptions of TV and print, their causal attributions to failure and success in learning from these media (i.e., PDC) as well as their PSE in learning from either TV or print. A week later, children either watched a silent TV film (TV condition), or read a textbook story with a comparable content (print condition). Findings revealed that children attributed more realism to TV than to print. They also reported a higher PSE for TV than for print. Children mostly attributed internal causes (i.e., ability and effort) to the learning success with print. External causes (i.e., difficulty and ease) were attributed to learning success with TV. In the print condition, children reported a higher AIME and scored higher on the achievement test. In addition, PSE and AIME were positively related in the print condition, while correlating negatively in the TV condition. Altogether, these results supported Salomon’s hypothesis: If stimulus material is perceived as easy, individuals with higher PSE will invest less mental effort (AIME) and thus learn less.

In subsequent studies, Salomon and Leigh (1984) further investigated children’s preconceptions of TV and print to explore how they were related to ability, AIME, and learning. Similar to earlier results (Salomon, 1984), children perceived print material as more demanding resulting in higher AIME. Moreover, children’s ability and media type interacted: children with high abilities scored lower on achievement in the TV condition, while less able children showed the reversed pattern. This finding could be explained by the fact that abler children expended less AIME in print (because of higher PSE) than less able children. Salomon and Leigh (1984) also experimentally changed children’s preconceptions of the AIME they need to spend on the content of a medium. Findings showed that AIME was generally higher in the print than in the TV condition. Moreover, AIME was higher in the learning than in the fun conditions established by instructions – with this increase being higher in the TV than in print condition. These findings support the assumption that preconceptions and image of a stimulus (PDC) influence AIME.

Beentjes (1989) aimed to replicate the findings of Salomon and colleagues with a Dutch sample of sixth-graders. In contrast to previous work, he did not implement an experimental manipulation but used questionnaires. Again children reported in general higher AIME for books than for TV programs. However, in contrast to Salomon’s findings, children of the TV condition did not generally report higher PSE. More specifically, the topic became relevant: e.g., information about sports was reported to be more easily learned by TV, while print was better for English language or Math. Coherent with Salomon’s findings, children attributed a higher realism to TV than books. Children also attributed the understanding of TV (vs. print) contents less to internal causes (i.e., effort and ability) and were more likely to attribute the failure to understand information from TV (vs. print) to external causes (i.e., difficulty of the material). Furthermore, the study of Beentjes (1989) could neither replicate the negative correlation of AIME and PSE for TV nor the positive correlation of AIME and PSE for print.

Cennamo (1992) explored learners’ preconceptions regarding different learning outcomes through interactive video, TV, books, and computers. Similar to Beentjes (see above), results revealed an interaction of learning outcome and medium. For instance, participants reported more difficulty learning psychomotor skills and attitudes from books and computers than learning the same content from TV and interactive videos. The reverse pattern was observed for learning verbal information and intellectual skills.

### AIME in New Media

Literature explicitly focussing the effects of new media is rare. Rieh et al. (2012) found that individuals invested more AIME when searching for information in a library system relative to a web search engine, because their PSE was higher when using the web search engine and they perceived the web search engine as easier than the information search through the library system.

### “Absorption” of the AIME Concept and Research Question

As shown above, the concept of AIME as originally proposed by Salomon (1984) stopped occurring in the literature somewhere in the 1990s and is hardly found in the current scientific literature. This leads to the research question of why this occurred. It is possible that the original concept of AIME has been absorbed by other research constructs, given that a number of similar terms exist in the literature, all describing mental effort. Among them are *mental effort, attention, concentration, mental workload*, and *cognitive load*, for instance (e.g., Cennamo, 1993; Kirschner and Kirschner, 2012). However, another explanation could be that the concept of AIME has disappeared due to a lack of replicability, for instance (see also, Beentjes, 1989). To clarify were the framework of AIME might have diffused, we conducted a literature search on research articles that cite Salomon (1984).

## Method

### Procedure and Sample of Literature Search: 30 Years AIME

For an overview of 30 years of research a literature search was conducted on Tuesday, 13 October, 2015. This was supplemented by a further review of literature on 6 February 2018. Following previous literature reviews (e.g., Fox et al., 2009), the database Web of Science^®^ (WoS) was used. WoS is an online academic database which pertains to the ISI Web of Knowledge^1^. The database was not accessed from the network of the University of Wuerzburg, Germany, given that access to scientific articles from this university is foremost restricted to research conducted after 1991. To overcome this shortcoming, WoS was accessed from the network of the Leibniz Institute for Psychology Information^2^ in Trier, Germany, which hosts the databases PSYNDEX and has thus extensive access to psychological scientific literature.

**FIGURE 1:**
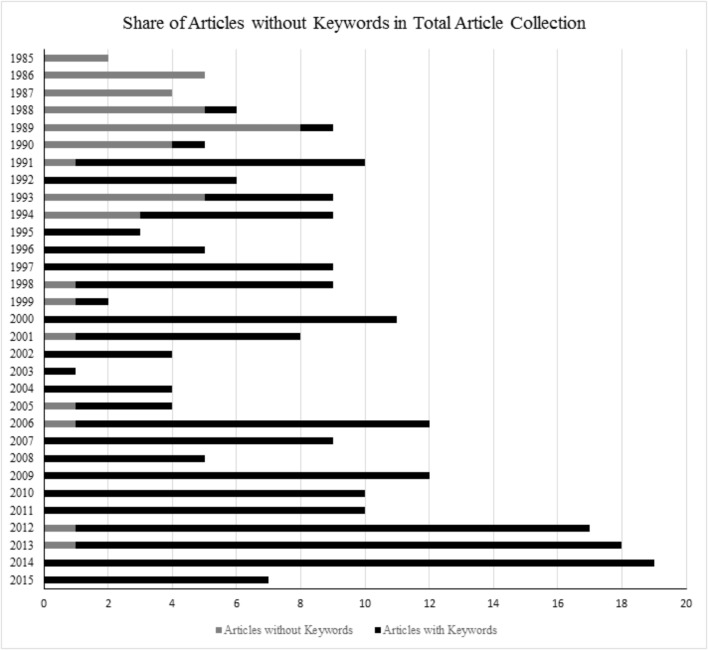
Number of articles by publication year citing Salomon (1984) in WoS (October 13, 2015).

In WoS, the database *Web of Science Core Collection* was selected and the function *cited reference search* was used to find articles that cite Salomon’s key article (1984). Every database reference citing this article was included in our selection. This search yielded 244 articles published between 1985 and 2015. The supplementing research from 2015 until 2018 revealed 30 more articles. We then content-analyzed (Mayring, 2000; Ramsenthaler, 2013) the keywords of these articles. Whenever possible, we used the author keywords for our content analysis. When the article provided no author keywords, we used the keywords provided by WoS. Of these articles, however, 44 had to be excluded as neither the article nor WoS provided keywords (see **Figure 1**).

For the remaining 200 articles, we extracted 1238 keywords (including duplications) of which 722 were distinct keywords (without duplication, see also **Tables 1**, **2**). **Table 3** in the appendix gives an overview of the additional search until the beginning of 2018. Having content-analyzed the keywords, these were described under generic terms, which, in turn, were assigned to content clusters. The content-analysis was performed by three academic psychologists proceeding inductively. Whenever discrepancies emerged between the experts to which cluster a keyword should be assigned, these discrepancies were extensively discussed and a final decision based on a consensus was made. Each keyword was exclusively assigned to one cluster.

**Table 1:** Content clusters based on content analysis of keywords.

Content cluster	Total number of			
	
	Keywords (incl. duplications)	Distinct keywords (no duplications)	Journal articles	Selected distinct journal articles within cluster
Affect and motivation	181	92	87	10
Application fields	125	96	80	10
Cognition and learning	214	113	112	10
Education and teaching	148	69	94	11
Learning with media technology	66	52	43	12
Media technology	266	129	123	12
Methods	124	96	77	10
Rest	114	75	78	–
Total	1238	722	–	31


**Total number of keywords N = 1238. Total number of distinct keywords N = 722. Total number of journal articles providing keywords N = 200.**

**Table 2:** Clusters, generic terms and keywords.

Content cluster	Generic term	Distinct keywords (examples)
Affect and motivation	Behavior	Expectancy, skill acquisition
	Emotion and affect	Impulsivity
	Motivation and goals	Goals, motivation, regulatory mechanism, work motivation
	Self-efficacy	Self-efficacy, self regulation
Application fields	Educational system	Academic performance, elementary-school
	Group	Peer models
	Health	Respite care
	Science	Mathematics, physics
	Violence and aggression	Aggression
	Working environment	Staff training
Cognition and learning	AIME	Investment, mental effort
	Attention and cognition	Attention, spatial skills
	Control	Control theory
	Experimental procedures	Ambiguity
	Imagination and cognitive representations	Children’s imagination
	Social cognitive theory	Social cognitive theory
	Task characteristics	Complex decision-making
Education and teaching	Instruction	Instruction, strategy instruction
	Language	Words
	Learning and teaching strategies	Effort attributional feedback, feedback, strategies
	Performance and achievement	Performance, task performance, achievement, children’s achievements, expert performance, writing achievement
Learning with media technology	E-Learning	Online learning
	Instructional media	Computer-assisted-instruction, instructional design, design of instructional hypermedia, design of instructional multimedia
	Learning environments	Adaptive learning environments
	Learning with films and videos	Video-based training
	Learning with media	Computer based training, media based learning
	Learning with multimedia	Multimedia learning
Media technology	Audio	Radio
	Gaming	Action videogames, computer games
	Hypermedia	Hypermedia
	Print	Printed Stories, text
	Specific media content	Visual information, technology-enhanced storybooks
	TV and Film	Television
Methods	Analysis	Predictors, theoretical analysis, contextual variables, experimental design, formal and informal theorizing
	Sample characteristics	Children, college students, preschoolers, skills, experience, family patterns, low income families
Rest		e.g., agreement, at-risk, choice, meanings, form, design, orientation, quality, utility, spiritual


**Table 3:** Content clusters based on updated content analysis of keywords until February 2018.

Content cluster	Total number of			
	
	Keywords (incl. duplications)	Distinct keywords (no duplications)	Journal articles until 2015	Journal articles from 2015 until 2018
Affect and motivation	211	114	87	16
Application fields	132	101	80	6
Cognition and learning	243	133	112	16
Education and teaching	163	75	94	12
Learning with media technology	74	57	43	7
Media technology	288	141	123	17
Methods	133	103	77	7
Rest	152	141	78	18
Total	1396	835	–	–


**Total number of keywords N = 1396. Total number of distinct keywords N = 835. Total number of journal articles providing keywords N = 230*.*

In a next step, ten different articles of each content cluster were selected from the 30 years of research period (1985–2015) limiting the analysis to studies published between 1985 and 2015. The rationale behind this procedure was to be able to describe the content clusters relating to AIME in more detail based on the articles that provided the keywords. To make a choice which articles to select, the IF of the journals in which the articles had been published was applies applied as a selection criterion. Although the validity of the IF has been criticized (e.g., Vanclay, 2012), the IF is still the most commonly used assessment aid when determining the quality of a journal (Garfield, 2003; Dong et al., 2005). The journal’s 2-years IF from the year 2014 as provided by the Thomson Reuter’s Journal Citation Reports^®^ Social Science Edition and Science Edition (Reuters, 2015) was used. The final selection comprised the 10 articles which yielded the highest journal IFs. For nine journals, Thomson Reuter’s Citation Reports^®^ did not yield an IF. Hence, articles published in these journals (*n* = 11) were not considered for the selection. In some cases (*n* = 14), it was not possible to make a clear choice of ten articles because after picking these, there were articles left which had been published in the same journals as the past picked article. In these cases, all articles were included in the selection until an article with a next lower journal IF was encountered. The selection of the journal articles yielded a total of 31 journal articles. The selected articles were distinct within each cluster; between the clusters, however, articles could double, as it was possible that the keywords of one article were assigned to different content clusters. In sum, *n* = 21 distinct articles remained.

## Results

### Content Clusters Resulting From Content Analysis

Based on the content analysis of the keywords, seven content clusters were established. Assigning research to these content clusters offers first insights regarding the research areas most intensively referring to AIME. These were as follows: *affect and motivation*, *application fields*, *cognition and learning*, *education and teaching*, *learning with media technology, media technology*, and *methods*. In additions, there was one category labeled *rest* to which all keywords were assigned that were vague or ambiguous in meaning and could not be clearly assigned to one category. See **Table 1** for the number of keywords (duplications and distinct), corresponding articles, and selected journals within the clusters.

### Description of Relevant Literature in Content Clusters

In this section each identified cluster will be described in more detail. Keywords and generic terms that relate to the clusters will be presented by briefly introducing the respective research articles (see **Table 2**). As the majority of the selected works provided keywords that were assigned to more than one content cluster, we refer to the same works at various points.

### Cluster: Affect and Motivation

#### Generic Term: Affect and Emotion

##### Keyword: impulsivity

Greenfield (2009) referred to the Flynn effect (Flynn, 1987) and discussed which effects informal learning environments such as TV, video games, and internet have on intelligence and cognitive abilities. The author pointed out that visual media foster the development of visual-spatial skills and iconic representation. However, she also pointed out that these fast-paced media do not allow the individual to rest and reflect about the content and can lead to a higher (intellectual) impulsivity.

#### Generic Term: Behavior

##### Keyword: expectancy

Vancouver et al. (2008) investigated the relationship between SE and motivation. Thereby, they referred to SE as a specific kind of expectancy which influences an individual’s motivation to accomplish a task or a goal.

###### Keyword: skill acquisition

The findings of Vancouver et al. (2001) that besides its positive effects, SE can also have a negative influence on subsequent performance are important for skill acquisition. Based on their empirical findings, it followed that when a person’s SE rises, he might not always enhance performance and acquire better skills but might also invest less effort in a task, given that he feels being fully capable of accomplishing it.

#### Generic Term: Motivation and Goals

##### Keywords: goals, motivation

Vancouver et al. (2008) suggested that the type of goal processes (i.e., goal choice and goal planning) affect the relationship between SE and motivation. They showed that SE is positively associated with motivation in goal-choice processes, given that individuals prefer anticipating an easy goal accomplishment relative to a difficult goal accomplishment or even failure. However, when participants plan for accepted goals, SE and motivation are negatively related because accepted goals demand more effort than anticipated easy goals. Based on these findings, the authors postulated a non-monotonic discontinuous association between SE and motivation.

##### Keywords: regulatory mechanism, work motivation

Vancouver et al. (2001) examined SE as a regulatory mechanism that influences the amount of resources and individual will invest in accomplishing a goal. The findings of Vancouver et al. (2001) had implications for work motivation. Contrary to the propositions of SE theory (Bandura, 1982), the authors claimed that SE, personal goals, and performance are not always positively related to each other. More specifically, using a within-subjects design, Vancouver et al. (2001) showed that past performance positively affects SE, while SE does not necessarily have a positive effect on subsequent performance. They explained the potential negative effect of SE on subsequent performance with a person’s complacent belief that he will master the task. Thus, the person might show less effort, given that he allocates resources to master goals that have not been accomplished, yet. However, Vancouver et al. (2001) also argued that SE can exhibit a positive relationship with subsequent performance when SE increases a person’s likelihood of choosing a difficult goal.

#### Generic Term: Self-Efficacy

##### Keyword: self-efficacy

Schmidt and DeShon (2010) examined how performance ambiguity moderates the relationship between SE and performance. They found that when performance ambiguity was high, SE and subsequent performance were negatively associated, while SE and performance yielded a positive correlation under conditions of low performance ambiguity. Vancouver et al. (2008) examined the relationship between SE and motivation and found evidence for a non-monotonic and discontinuous relationship. The process of goal choice evoked positive discontinuity, while the process of goal planning evoked a negative effect. In a later article, Vancouver (2012) criticized Bandura’s approach of theorizing using examples from SE theory and social cognitive theory. Vancouver (2012) argued that Bandura would describe SE concepts in “informal” (p. 466) natural language with the use of fallacious rhetorical means and contradict formerly posited theoretical statements.

##### Keyword: self-regulation

Vancouver et al. (2008) and Schmidt and DeShon (2010) referred to SE as a crucial component of self-regulation which is related to an individual’s pursuit of goals, allocated effort, and persistence.

### Cluster: Application Fields

#### Generic Term: Education System

##### Keyword: academic performance

Haimerl and Fries (2010) provided evidence for self-fulfilling prophecy effects in media-based learning which affect students’ academic performance. In particular, when students had high expectations regarding an instructional medium’s quality, they showed an enhanced academic performance. This relationship was further moderated by learning content relevance such that when content relevance was moderate, explicit positive information about the medium’s quality increased students’ performance. When learning content relevance was high, however, students unexpectedly showed a better performance when the information on the instructional medium’s quality was explicitly negative.

##### Keyword: elementary school

Bong and Clark (1999) reviewed research on the relationship of academic SE and self-concept. The authors reported studies that involve subjects from elementary school (Eccles et al., 1993; Helmke and van Aken, 1995; Pajares and Valiante, 1999). In sum, the presented studies suggested that children develop a self-concept already in elementary school and are able to distinguish between their specific abilities in different academic domains (e.g., math, reading, music; Eccles et al., 1993). Moreover, previous achievement appeared to rather influence academic self-concept than vice versa in elementary school (Helmke and van Aken, 1995).

#### Generic Term: Health

##### Keyword: respite care

Neef et al. (1991) investigated the effectiveness of videotapes as instructional media in the field of respite care. Their results provided support that videotapes can be an alternative to printed training materials when combined with an individual remedial training. Presentation format (i.e., viewing the tape alone, with another person, or in a structured group) did not affect the results.

#### Generic Term: Group

##### Keyword: peer models

In his overview article, Schunk (1991) presented findings on the relationship of SE and academic performance. He pointed out that that perceived SE can be influenced by peer models, that is, an individual can obtain information on the possibility to master a task by observing similar peer models who accomplish the task (Schunk, 1989). Thereby, the observed person is of importance, that is, the more similar the observed person is to oneself the more SE is increased (Schunk and Hanson, 1985). In a later article, Schunk and Zimmerman (1997) reviewed theories on the development of self-regulatory skills. Thereby, they emphasized observational learning from peers.

#### Generic Term: Science

##### Keyword: mathematics

Bong and Clark (1999) reviewed empirical findings suggesting that academic SE is a better predictor of achievements in a specific field (including math) than academic self-concept (Pajares and Miller, 1994; Marsh et al., 1997).

##### Keyword: physics

In his review article, Kozma (1991) described the advantages of learning with TV for specific subjects (e.g., physics). He summarized research that suggests that animated pictures can provide information on the dynamics of a model that fosters a learner’s understanding (Holland et al., 1986). Due to their visual stability, texts cannot provide this information. In a similar vein, Kozma discussed that learning with computers by collecting real time data and displaying it can enhance understanding of physics (Brasell, 1987).

#### Generic Term: Violence and Aggression

##### Keyword: aggression

Valkenburg and van der Voort (1994) presented findings on TV consumption and children’s creative thinking and daydreaming. In sum, they suggested that exposure to violent TV programs can foster aggressive daydreaming and fantasies. However, they also called for more research that systematically investigates this relationship. In a further review article, van der Voort and Valkenburg (1994) summarized again research on the influence of TV on children’s fantasy play. They found that TV programs that contain a lot of violence and aggression decrease fantasy play. On the other hand, however, TV programs with no violence did not increase children’s fantasy play.

#### Generic Term: Work Environment

##### Keyword: staff training

Neef et al. (1991) examined the effectiveness and aptitude of videotapes for staff training in the field of respite care provision. Their findings supported videotapes as an effective method for staff training when combined with an individual remedial training.

### Cluster: Cognition and Learning

#### Generic Term: AIME

##### Keywords: investment, mental effort

Valkenburg and van der Voort (1994) reviewed the influence of TV on children’s and adults’ daydreaming and creative imagination. Their review supported the assumption that TV viewing fosters daydreaming, while it decreases creative imagination. In the course of their literature review, they described different versions of the reductionist hypothesis which generally claims that TV consumption would decrease daydreaming. One version explained the reduction in daydreaming with the fact that TV presents the viewer with fantasies and imagination which have been produced by others (e.g., the director or the producer). Viewers then invest little mental effort to process these fantasies and, consequently, do not invest effort in creating their own fantasies and imaginations. However, Valkenburg and van der Voort also described work that is critical of the assumption that daydreaming is the product of a high AIME (Klinger, 1990). In addition, Greenfield (2009) stated that mental effort is required for scientific thinking. She proposed that fast-paced visual media do not foster reflection because they do not allow the individual to stop and carefully think about content (Gadberry, 1981). Instead, Greenfield proposed that no real-time visual media, such as print and books, which are perceived to be hard and thus require more mental effort (Salomon, 1984), are important to acquire reflection, critical thinking, inductive analysis, and imagination.

#### Generic Term: Attention and Cognition

##### Keyword: attention, spatial skills

Greenfield (2009) described the effects of informal learning environments consisting of visual real-time media (e.g., TV, video games, internet) on visual intelligence. She claimed that visual media, particularly video games, foster divided attention (e.g., Greenfield et al., 1994), which is considered a precondition of multitasking behavior. She further presented research that suggests that tasks are better learned in single-task conditions relative to multitasking conditions (Foerde et al., 2006). In his review, Kozma (1991) described research on learning media. Thereby, he explained how specific media characteristics can capture a learner’s attention and thus affect learning achievement. Greenfield (2009) further stated that visual real-time media can increase an individual’s visual-spatial skills. As an example, she described the research of Rosser et al. (2007) that demonstrated that playing video games can have a training effect on spatial skills which are required for the conduct of specific medical operations.

#### Generic Term: Control

##### Keyword: control theory

Vancouver (2012) criticized Bandura’s “informal” (i.e., verbal, p. 466) theorizing (e.g., Bandura and Locke, 2003) with a focus on the language Bandura uses. Vancouver (2012) contrasted Bandura’s description of the role of SE with the approach Powers (1973) took in his control theory which, in turn, Vancouver considered a “formal, computational approach” (p. 466). Vancouver also brought up Powers (1991) critique of Bandura’s theorizing. Schmidt and DeShon (2010) drew on Powers (1973) control theory as well and extended the work of Vancouver et al. (2001, 2008) by demonstrating a negative relationship between SE and subsequent performance when performance ambiguity was high. When performance ambiguity was low, SE and performance were positively associated.

#### Generic Term: Experimental Procedures

##### Keyword: ambiguity

Schmidt and DeShon (2010) used performance ambiguity to explore the relationship between SE and task performance. To this end, they used an experimental paradigm that consisted of an anagram task. In the instruction, participants were informed that the anagram task had different numbers of possible solutions (ranging from no solutions to more than one solution). The number of solutions served as operationalization of performance ambiguity.

#### Generic Term: Imagination and Cognitive Representations

##### Keyword: children’s imagination, imagination

Valkenburg and van der Voort (1994) reviewed the influence of TV on children’s and adults’ daydreaming and creative imagination. Their results support the assumption that TV viewing fosters daydreaming, while it decreases creative imagination. However, the authors did not find evidence for a clear causal relationship.

#### Generic Term: Social Cognitive Theory

##### Keyword: social cognitive theory

Vancouver et al. (2001) criticized core assumptions of social cognitive theory (Bandura, 1989) by suggesting that SE could undermine performance when individuals consider their goal as almost accomplished. In contrast, social cognitive theory proposes that SE positively relates to motivation and performance (Bandura and Locke, 2003). In a later comment, Vancouver (2012) responded to Bandura’s critique of his work (Bandura, 2012) and again criticized Bandura’s theorizing with respect to SE.

#### Generic Term: Task Characteristics

##### Keyword: complex decision making

According to Vancouver et al. (2001), the relationship between SE and performance is influenced by personal goals chosen in a decision making process. The authors found that SE and personal goals exhibited a negative relationship with task performance.

### Cluster: Education and Teaching

#### Generic Term: Instruction

##### Keyword: instruction

Schunk (1991) described the relationship between SE and academic motivation. Thereby, he reviewed how variations in instructions (e.g., general vs. specific goals, distant vs. proximal goals, use of specific strategies) influence SE and subsequent goal accomplishment and skill acquisition.

##### Keyword: strategy instruction

Schunk and Zimmerman (1997) reviewed theories that explain the development of self-regulatory skills (e.g., time management, rehearsal, attention to instructions) and specifically focused on observational learning through modeling. Schunk and Zimmerman (1997) described results which showed that strategy instructions for a specific tasks presented by a model enhances students’ SE and performance and might even generalize to other tasks (e.g., Graham and Harris, 1989).

#### Generic Term: Language

##### Keyword: words

Wright et al. (2001) measured children’s television viewing of informative child-audience (i.e., educational) and general-audience programs, and related it to the children’s academic achievement and skills. Among other measures, academic achievement was assessed by word recognition of simple objects and more complex concepts. Their results showed that children who were exposed to child-audience TV programs had better prereading and reading skills given that they recognized more words in a reading and receptive vocabulary test.

#### Generic Term: Learning and Teaching Strategies

##### Keyword: feedback

Schmidt and DeShon (2010) demonstrated that the relationship between SE and performance is moderated by performance ambiguity. They found that when performance ambiguity was high, SE and performance were negatively related, while when performance ambiguity was low, they were positively related. To investigate this effect, Schmidt and DeShon (2010) used different kinds of feedback (i.e., the number of possible solution sin their experimental task) to manipulate performance ambiguity.

##### Keyword: effort attributional feedback

Schunk (1991) reviewed findings on the effect of effort attributional feedback (i.e., feedback that explicitly links performance to one’s effort) on SE and performance. The author presented evidence that effort attributional feedback can enhance motivation, SE, and performance (Schunk, 1982; Schunk and Cox, 1986). In an exploratory review article, Bong and Clark (1999) compared academic self-concept and SE. The authors thereby also referred to the studies of Schunk and colleagues (e.g., Schunk, 1982; Schunk and Hanson, 1985; Schunk and Cox, 1986) which suggested that instructional programs, such as effort attributional feedback, positively affect academic SE beliefs. These academic SE beliefs, in turn, causally and positively predict students’ engagement in achievement-related behavior and can thus increase performance.

##### Keyword: strategies

Hooper and Hannafin (1991) described design strategies of instructional media that consider the learner’s cognitive processes. Thereby, the authors specifically referred to five cognitive phases (i.e., retrieving, orienting, presenting, encoding, sequencing) as proposed by Hannafin and Rieber (1989) and presented design strategies for instructional media relating to the cognitive phases.

#### Generic Term: Performance and Achievement

##### Keyword: achievement

Wright et al. (2001) found a positive effect on children’s early television viewing of child-audience informative programs (in this case *Sesame Street*) on their subsequent academic achievements. These were assessed by reading and math tests, receptive vocabulary tests, and school readiness.

##### Keyword: children’s achievement

Schunk and Zimmerman (1997) presented an overview on the development of children’s self-regulatory skills with a focus on observational learning through modeling. The authors showed that learning by observing a model positively affects children’s achievements.

##### Keywords: expert performance

Feldon (2007) reviewed research on teaching and teacher education and focused on the role of dual processing models and cognitive load in teacher performance. In line with the dual process model, Feldon provided evidence that with increasing expertise, teachers develop routines that enable them to show more effective instructions and classroom management (Leinhardt and Greeno, 1986). That is, they were more able to distinguish between relevant and irrelevant cues in class due to automated procedures that leave working space for the processing of new events in the classroom (Kagan and Tippins, 1992; Allen and Casbergue, 1997). However, Feldon also pointed of the downsides of automaticity in teaching, for instance, that teachers were hardly able to change their way of teaching when it has become an automated routine (Doyle and Redwine, 1974). Moreover, novice teachers were likely subject to cognitive biases (i.e., expectancy effects/Pygmalion effects), as due to a high cognitive load, effortless and unconscious heuristics were more likely to occur (Babad, 1985).

##### Keyword: performance

Greenfield (2009) described how the exposition to visual, fast-paced media (e.g., TV, video games, and internet) can affect an individual’s cognitive performance measured by visual IQ. The author stated that visual media can foster visual-spatial skills and iconic representations, which, in turn, can provide beneficial effects for specific action, such as surgeries requiring spatial imagination and navigation (Rosser et al., 2007). However, the author also spoke critically of the fast-pace visual media and pointed out that they can decrease reflection and increase impulsivity because they do not leave time to contemplate over a specific content (Gadberry, 1981). Thus, Greenfield (2009) recommended a well-balanced use of real-time and no real-time (i.e., print) media. Schunk (1991) described how SE and academic performance relate to each other and how their relationship is influenced by personal variables (i.e., goal setting and information processing) and situational variables (models, attributional feedback, and rewards). In a later review article, Schunk and Zimmerman (1997) presented evidence that observational learning positively affects children’s SE, motivation, and (academic) performance.

##### Keyword: task performance

Vancouver et al. (2001) pointed out that SE can be differently related to task performance. In two experiments, they found that past performance positively predicted SE, whereas SE can negatively affect subsequent performance. That is, when a person is too confident about accomplishing a task so that he puts no more effort in the task. Furthermore, SE can also positively relate to subsequence performance when a person selects a difficult goal.

##### Keyword: writing achievement

Schunk and Zimmerman (1997) described literature on the association between the development of children’s self-regulation processes and learning by observing a model. The authors gave examples for a positive relationship between these variables by citing work that examined children’s achievements in paragraph writing after obtaining instructions by an adult model (Schunk and Swartz, 1993). Moreover, children had higher writing achievements when they were given a process-goal (i.e., learning) and feedback during their learning progress (Schunk and Swartz, 1993) compared to when they were provided a product (i.e., performance) or a general goal (i.e., control). Bong and Clark (1999) compared self-concept and SE in the field of academic motivation research. The authors concluded that specific task performances, such as writing achievements, are better explained by academic SE than academic self-concept (Pajares, 1996; Pajares and Valiante, 1999; Pajares et al., 1999).

### Cluster: Learning With Media Technology

#### Generic Term: Instructional Media

##### Keyword: computer-assisted instruction

Hooper and Hannafin (1991) presented an overview on how instructional media could be designed so that they fit with the learner’s cognitive processes and thus enhance learning outcomes. During their work, they also addressed computer-assisted instructions and proposed that these could enhance meaningfulness for the learner by personalization, that is, when they include specific information about the learner which has been previously assessed from a database (Anand and Ross, 1987).

##### Keywords: design of instructional hypermedia, design of instructional multimedia

Zahn et al. (2004) studied the effects of design principles that were derived from either hypermedia (i.e., user-friendly interfaces; hyperlinks are presented at the end of the respective video scenes in a small cluster) or multimedia (i.e., spatio-temporal contiguity; hyperlinks are sequentially presented in the medium close to the corresponding visual information) on students’ learning with an instructional hypervideo. Results showed that design principles did not affect students’ knowledge acquisition. Instead, knowledge acquisition was rather positively influenced by individual characteristics of navigation behavior within the hypermedia.

##### Keyword: instructional design

In his overview article, Feldon (2007) described research on cognitive models of information processing and related them to findings on teacher cognition and class teaching. Thereby, he focused on the dual-process model of cognition and points out the advantages and disadvantages of behavioral routines and fixed scripts in classroom teaching. As one example to include automaticity into teacher training, Feldon described the teacher training of Van Merriënboer (1997) labeled four-component instructional design (4C/ID) system. This training fostered automaticity for *recurrent* skills, that is, for skills that are very similar in a lot of situations and can thus be well automatized. As a result, cognitive load is held restricted and working memory is free for *non-recurrent* skills that have a consistent goal but differ between the concrete situations.

#### Generic Term: E-Learning

##### Keyword: online learning

Inglese et al. (2007) investigated how audiovisual TV interviews with authors (vs. print) affected the academic performance of native and non-native speakers. They found that when authors were visible in the TV condition, native and non-native speakers did not perform differently. However, when the author was non-visible (i.e., the print condition) non-native speaker performed worse. Inglese et al. (2007) explain their findings by the fact that in the audiovisual TV interview conditions students were more able to create a personal relationship with the respective author. Moreover, non-native speakers could be subject to cognitive overload when they have to process the instructions in a foreign language in the print condition and might thus show lower academic achievements. Based on these results, Inglese et al. (2007) proposed that audiovisual media can be embedded in online learning in academic settings and enhance learning outcomes, specifically those of non-native speakers.

#### Generic Term: Learning Environments

##### Keyword: adaptive learning environment

Gerjets et al. (2014) described how digital learning environments can be designed so that these do not impose cognitive over- or underload on the learner. To this end, the authors proposed to measure the learner’s cognitive load continuously using a passive Brain–Computer Interface approach so that digital learning environments can adapt to the amount of cognitive load and provide optimal learning conditions.

#### Generic Term: Learning With Films/TV

##### Keyword: video-based training

Neef et al. (1991) explored the effectiveness of video-based trainings for respite care providers considering also contextual factors (i.e., viewing the material alone vs. viewing it in a dyad or group). Their results provided evidence that video-based trainings can be an alternative to printed training materials. However, video-based training should be combined with individual remedial trainings.

#### Generic Term: Learning With Media

##### Keywords: computer based training, media based learning

Fries et al. (2006) showed that quality expectations influenced students’ quality ratings and actual achievements in media-based learning. When students expected to work with a high-end computer-based training program, they showed the highest learning achievements, while the expectation of ambiguous quality led to the lowest achievements. Students who did not have any expectations scored somewhere in between. The effect could be replicated when students were provided with an additional learning script which aimed to benefit a deeper processing. In a later study, again Haimerl and Fries (2010) found that explicit information (positive vs. negative) about an instructional medium’s quality triggered self-fulfilling prophecy effects and influenced students’ academic performance. Learning content relevance acted as a moderating variable. In their experiment, the authors used various fictitious web-based training programs with hypertext.

#### Generic Term: Learning With Multimedia

##### Keyword: multimedia learning

Bus et al. (2015) presented a review on research on the relationship between technology-enhanced storybooks and children’s literacy. They described findings that indicated that children have a better performance when learning with multimedia that present visual and verbal information in close temporal contiguity (e.g., Paivio, 2008; Takacs and Bus, 2013, April). Moreover, well-designed multimedia might be especially beneficial for susceptible children (Belsky et al., 2007) by drawing their attention and motivation to the storybooks. However, when pictures were rather decorative and did not illustrate specific content in the text, they did not positively contribute to children’s story comprehension and vocabulary learning (e.g., Korat and Shamir, 2007). In a further study, Inglese et al. (2007) showed that non-native speakers achieved a higher academic performance when they learned with multimedia presentations involving audiovisual TV interviews of authors. Inglese et al. (2007) concluded that a visual presentation of the author fosters the students’ forming of a relationship with him or her. Furthermore, a multimedia presentation might evoke less cognitive overload in non-native speakers because they do not have to invest a lot of cognitive effort to basic language processing but can allocate cognitive load to understand the presented material as a whole.

### Cluster: Media Technology

#### Generic Term: Audio

##### Keyword: radio

Valkenburg and van der Voort (1994) presented research on the relationship between TV viewing and children’s daydreaming and creative imagination. They reviewed studies that compare children’s number of novel elements in a story after being exposed to a TV or radio condition. To summarize, there were mixed findings: there was partial support for the claim that TV reduces children’s creative thinking (Greenfield et al., 1986), and that children’s creative thinking was further influenced by ethnicity, socioeconomic status, and story comprehension (Greenfield and Beagles-Roos, 1988). Other studies found no differences in children’s creativity (Runco and Pezdek, 1984), or mixed results (Vibbert and Meringoff, 1981).

#### Generic Term: Hypermedia

##### Keyword: hypermedia

Bus et al. (2015) summarized findings on the relationship between electronic storybooks and children’s literacy. They concluded that media including hypermedia may lead to a cognitive overload in children and thus decrease performance with respect to vocabulary tests and story comprehension (e.g., Trushell et al., 2003; Courage et al., 2015).

#### Generic Term: TV and Film

##### Keyword: television

Greenfield (2009) presented findings which indicate that fast-paced visual media including TV can increase visual cognitive skills. However, the author was also critical about the potential benefits these media may have and presented empirical evidence that they can also lead to divided attention (Greenfield et al., 1994). Divided attention, in turn, can decrease task-learning (Bergen et al., 2005). Valkenburg and van der Voort (1994) summarized findings on how TV consumption influences children’s and adults’ daydreaming and creative imagination. The authors came to the conclusion that TV viewing might foster daydreaming but hinder creative imagination. However, they emphasized that there is not enough evidence to claim a causal relationship. In a recent study, Nathanson et al. (2014) investigated how preschoolers’ television exposure relates to their development of executive functions. Their results showed that the time of TV exposure was negatively associated with the children’s executive functions. Moreover, the earlier children started viewing TV, the weaker their executive functions were. High-quality educational programs without commercial breaks positively related to the children’s executive functions, while educational cartoon viewing was negatively associated. Kozma (1991) reviewed findings on learning with media and also described research dealing with TV and learning. Kozma concluded that television viewers are attracted to TV rather by bottom-up processes as provided by visual information. Moreover, he referred to the findings of Salomon (1984) and explained that information learnt from TV might be shallower and less deeply processed. However, Kozma also pointed at the fact that when individuals are *instructed* to learn from TV, they retain more information and process there more deeply. Furthermore, the author mentioned the benefits of TV, that is, the visual component which can be easily remembered. Nevertheless, he was also critical of the fast-paced visual presentation of TV which requires certain knowledge about the topic. Because the viewer has only little time to understand and process the information, novices might have problems following the fast rate of information presentation.

#### Generic Term: Gaming

##### Keywords: action video games, computer games

Greenfield (2009) summarized research on the effects of informal learning environments comprising real-time visual media on cognitive ability. She presented results that support a positive effect of video games on spatial skills (De Lisi and Wolford, 2002). However, she also pointed at the downsides of video games and computer games by describing negative consequences such as increased levels of aggression, decreased prosocial behavior, and desensitization (Carnagey et al., 2007).

#### Generic Term: Print

##### Keyword: printed stories

Valkenburg and van der Voort (1994) reviewed findings on the relationship between the amount of TV consumption, daydreaming, and creative imagination. They presented research which indicates that there are no or only little significant differences in children’s creative imagination between print, audio, and video (Meline, 1976; Kerns, 1981).

##### Keyword: text

Kozma (1991) summarized research on learning with media. He also referred to books as learning media and pointed at their characteristic of providing information in a stable way. Readers can decide how fast they want to process information and it is easily possible to stop at one difficult passage or to reread a passage. Moreover, books require building mental models out of the text. Print further offers the possibility of using specific text strategies (e.g., providing a title with a paragraph) that help the reader to recall context knowledge and to remember contents of the text passage better (e.g., Bransford and Johnson, 1972).

#### Generic Term: Specific Media Content

##### Keyword: technology-enhanced storybooks

Bus et al. (2015) reviewed research on the relationship between technology-enhanced storybooks and children’s literacy. They concluded that these media can both positively and negatively affect children’s story retention. The most important factor is whether technology-enhance storybooks present information that are compatible with information processing.

##### Keyword: visual information

Kozma (1991) provided a summary of research dealing with media. He thereby drew on different types of media and described the role of visual information in learning.

### Cluster: Methods

#### Generic Term: Analysis

##### Keyword: contextual variables, experimental design

Neef et al. (1991) included contextual variables when exploring the effectiveness of a video-based training in the field of respite care provision. The contextual variables referred to (a) viewing the videotape alone, (b) viewing the videotape with a partner, and (c) viewing the videotape within a group. Neef et al. (1991) found evidence that video-based trainings can be effective. However, contextual variables did not significantly influence training effectiveness. Moreover, they used experimental design to explore the effects of video-based trainings of respite care providers. They specifically employed a within-subjects Latin square design to test their effects.

##### Keyword: formal and informal theorizing

Vancouver (2012) criticized Bandura’s approach of theorizing (e.g., Bandura, 1989) and called it an “informal” approach which is “more vulnerable to interpretations errors and ambiguities” (p. 469). Vancouver considered the main reason for that in the fact that Bandura used natural language for his theorizing and contrasted Bandura’s approach with the “formal (i.e., mathematical or computational)” (p. 469) approach Powers (1973) used in his control theory.

##### Keyword: predictors

Valkenburg and van der Voort (1994) reviewed the research status on the relationship between TV consumption and creative imagination and daydreaming. They presented a longitudinal study by Singer et al. (1984) which found that children’s total viewing in the 1st year and the exposure to action and adventure films negatively predicted subsequent creativity scores.

##### Keyword: theoretical analysis

Vancouver et al. (2001) examined the relationship between SE and past as well as subsequent performance. Based on a theoretical analysis of the literature, specifically control theory (Powers, 1973), they claimed that SE can also be negatively related to subsequent performance if a person complacently feels that he will master a task and thus does not put much effort in accomplishing it. Based on their findings, they questioned one of the core propositions of social cognitive theory (Bandura, 1989).

#### Generic Term: Sample Characteristics

##### Keyword: children, experience

In her summary article, Greenfield (2009) reviewed findings relating to the effects of fast-paced visual media on spatial skills. Thereby, she referred to many studies involving children as participants (e.g., Kagan, 1965; Kagan et al., 1966; Gadberry, 1981). Moreover, she pointed out that previous experience with fast-paced visual media, more specifically, video games, can predict skills that are not directly related to the video games (e.g., laparoscopic skills in medicine, Rosser et al., 2007).

##### Keyword: family patterns

Valkenburg and van der Voort (1994) reviewed research relating to the association between viewing TV and children’s daydreaming and creative imagination. Among other variables, they presented findings suggesting that family patterns (i.e., parental orientation, family lifestyle, parental values of resourcefulness) predict creativity scores (Singer et al., 1984).

##### Keyword: preschoolers

Nathanson et al. (2014) explored how TV exposure during preschool years is associated with development of executive functions in children. The authors found different relationships between TV exposure and the development of executive functions. Type of program and age at which children start television viewing were influencing factors.

##### Keyword: skills

Wright et al. (2001) showed that early television viewing of child-audience informative programs enhanced children’s academic skills. Moreover, children’s skills influenced their television viewing behavior. More specifically, preschoolers with higher skills chose a larger amount of child-audience informative programs, while preschoolers with lower skills tended to watch more general-audience programs. Based on these findings, Wright et al. (2001), concluded that it is foremost the specific program content that affects the development of children’s academic achievements and skills. The authors also found evidence that the influences of early TV watching appear to be stable.

### Journals of Selected Research Articles

**FIGURE 2:**
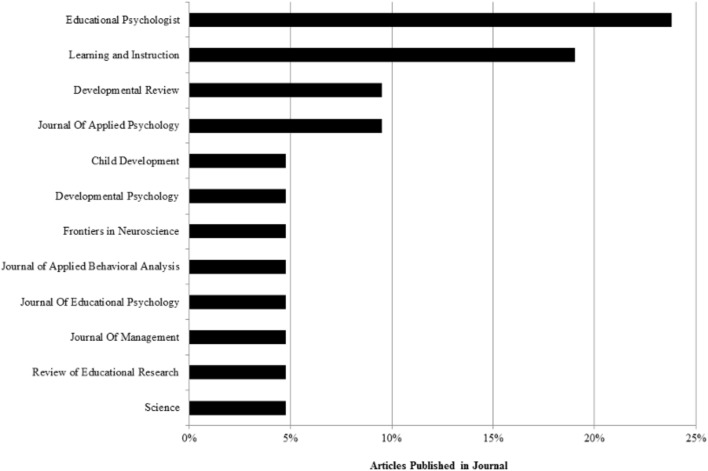
Peer-reviewed journals in which selected distinct research articles (*n* = 21) were published.

Next, we analyzed the journals in which the selected works had been disseminated (see **Figure 2**). To summarize, most of the described journal articles had been published in the realm of educational and instructional psychology. More specifically, almost a quarter (23.8%) had been published in *Educational Psychologist*, followed by 19% which had been published in *Learning and Instruction*. Both journals focus on research covering issues pertaining to education and educational psychology across all ages. Next, research had been published in *Developmental Review* and the *Journal of Applied Psychology* (each 9.5%). The former journal covers research dealing with developmental psychology, while the latter is a journal that covers all issues concerning practical implications of psychological theories. Thereby, situational factors and situations are of specific importance. The remaining articles (38.1%) were published in a variety of journals that cover the fields of developmental psychology (19%), neuroscience (4.8%), behavioral and clinical psychology (4.8%), managerial and organizational psychology (4.8%), and general science (4.8%).

## Discussion

Our analysis demonstrated that the concept of AIME (Salomon, 1984) is referred to in different areas of research. Based on the content analysis of the keywords, most of them cover educational psychology, teaching, cognition, and learning. In particular, the concept of AIME was cited in relation to instructions, effort feedback, learning strategies, and learning outcomes, such as task performances and (academic) achievements. This is further reflected in the content clusters *learning with media technology* and *media technology* given that most studies that were assigned to these clusters mentioned AIME in the contexts of learning environments (offline and online learning), and learning with different kinds of media (e.g., multimedia, computer-based trainings, video-based trainings, hypermedia). Thereby, the concept of AIME does not only appear in relation to print and TV as in Salomon’s (1984) original study but is also often used in the contexts of computer, hyper- and multimedia as well as (adaptive) e-learning environments. These findings indicate that AIME is relevant for new media too, and is not restricted to the comparison between text and TV, only. However, none of these studies directly worked with the concept of AIME which suggests that the relevance of AIME as a research construct has decreased over time. In addition, research referred to AIME in the contexts of attention and cognition, namely when mental effort or investment were described but also when it came to attention and spatial skills. All these results fit with the analysis of the journals in which the works were selected, as they pertained mostly to the field of educational and instructional psychology. Moreover, these results are reasonable, since one starting point of Salomon’s (1984) research was to investigate the effects of different media on children’s learning outcomes. Strikingly, AIME was also mentioned in relation to imagination, social cognitive theory, decision making, theories of motivation, and regulatory mechanisms. This is interesting, given that Salomon’s concept does not directly refer to these research areas. However, at second glance, these studies cite AIME when they describe SE (Bandura, 1982), which is also one component that affects AIME (Salomon, 1984).

With regards to the cluster *application fields*, AIME was often referred to in the context of educational systems, academic performances (both general and specific), and education institutions (e.g., elementary school). Beyond that, AIME was mentioned in the fields of health care and staff training. These findings suggest that the concept of AIME does not only play a role in research related to learning and performance in schools, but also in research on adult education and employee trainings. Finally, AIME was cited with regards to methods. Thereby, experimental design, formal and informal theorizing, and contextual variables were of importance. Furthermore, studies citing AIME used specific samples which predominantly included children (both preschoolers and school children) but also adults. These results indicate that references to the concept of AIME are not restricted to children’s learning but transfer to adults’ learnings and achievements as well.

### Absorption of AIME or Still Single Concept?

One goal of this research was to answer the question of whether and where the concept of AIME has diffused. As proposed, our results suggest that AIME has been at least partly absorbed by other research theories, such as cognitive load theory (see also, Kirschner and Kirschner, 2012). However, our analyses also indicate that AIME may still exists as a single concept given that also recent studies refer to it (e.g., Rieh et al., 2012; Nathanson et al., 2014; Bus et al., 2015), at least for historical reasons. Nevertheless, the framework of AIME is seldomly applied in current research anymore (for an exception, see Rieh et al., 2012).

### Meaning of AIME in Current Educational and Media Psychological Research and Directions for Future Research

Based on our findings, it appears that the single concept of AIME has only a little meaning for current educational and media psychological research. However, it has not fully vanished given that many studies still cite Salomon (1984). AIME is also referred to in current studies that investigate learning with new media, indicating that the impact of this concept reaches beyond research on text and TV (e.g., Korat and Shamir, 2007; Haimerl and Fries, 2010). This suggests that there is still an interest for AIME in educational and psychological research. Thus, future studies could increasingly make use of AIME and use it as a basis for their research. In particular, it would be interesting to investigate the amount of mental effort individuals are consciously willing to expend when they learn information from digital and mobile media, such as smartphones and tablet PCs. While AIME seems to focus on consciously deciding to invest more mental effort. Most of the theories that seem to have absorbed this classic concept focus on cognitive processes that are unconscious. Between those two approaches there is an interesting field where willingness to apply mental effort is comparable with measurable cognitive investment. While the field of research seems to focus on measurable de facto cognitive effort the willingly intended AIME seems to fade away as a research heuristic. This question is of large importance given that a lot of schools already use digital media (e.g., BITKOM, 2015) and that mobile learning is considered a mega trend (Zukunftsinstitut, 2016).

## Author Contributions

FS was head of the contribution and managed and developed the conception and design of the article; was responsible for data acquisition and interpretation;drafted the work and revised it for important intellectual content; gave final approval of the version to be published; and agreed to be accountable for all aspects of the work. CH contributed substantially to the conception, design, acquisition and interpretation of the data; drafted the article and revised its content; gave final approval for the version to be published; and agreed to be accountable for all aspects of the work. DA contributed to the design of the article, revised the contribution critically, gave final approval of the version to be published, and agreed to be accountable for all aspects of the work. AC also contributed substantially to the conception, design, acquisition and interpretation of the data; drafted and revised the content; gave final approval for its publication; and agreed to be accountable for all aspects of the work.

## Conflict of Interest Statement

The authors declare that the research was conducted in the absence of any commercial or financial relationships that could be construed as a potential conflict of interest.

## Footnotes

1 http://www.webofknowledge.com/

2 http://www.zpid.de/

## Abbreviations

AIME: amount of invested mental effort
IF: impact factor
PDS: perceived demand characteristics
PSE: perceived self-efficacy
SE: self-efficacy
